# A Major Root Architecture QTL Responding to Water Limitation in Durum Wheat

**DOI:** 10.3389/fpls.2019.00436

**Published:** 2019-04-10

**Authors:** Samir Alahmad, Khaoula El Hassouni, Filippo M. Bassi, Eric Dinglasan, Chvan Youssef, Georgia Quarry, Alpaslan Aksoy, Elisabetta Mazzucotelli, Angéla Juhász, Jason A. Able, Jack Christopher, Kai P. Voss-Fels, Lee T. Hickey

**Affiliations:** ^1^Queensland Alliance for Agriculture and Food Innovation, The University of Queensland, Brisbane, QLD, Australia; ^2^Laboratory of Microbiology and Molecular Biology, Faculty of Sciences, Mohammed V University, Rabat, Morocco; ^3^International Center for Agricultural Research in the Dry Areas, Rabat, Morocco; ^4^CREA - Genomics Research Centre, Fiorenzuola d’Arda, Italy; ^5^School of Science, Edith Cowan University, Joondalup, WA, Australia; ^6^School of Agriculture, Food & Wine, Waite Research Institute, The University of Adelaide, Urrbrae, SA, Australia; ^7^Leslie Research Facility, Queensland Alliance for Agriculture and Food Innovation, The University of Queensland, Brisbane, QLD, Australia

**Keywords:** root angle, seminal roots, root architecture, GWAS, QTL, haplotype, drought adaptation

## Abstract

The optimal root system architecture (RSA) of a crop is context dependent and critical for efficient resource capture in the soil. Narrow root growth angle promoting deeper root growth is often associated with improved access to water and nutrients in deep soils during terminal drought. RSA, therefore is a drought-adaptive trait that could minimize yield losses in regions with limited rainfall. Here, GWAS for seminal root angle (SRA) identified seven marker-trait associations clustered on chromosome 6A, representing a major quantitative trait locus (*qSRA-6A*) which also displayed high levels of pairwise LD (*r*^2^ = 0.67). Subsequent haplotype analysis revealed significant differences between major groups. Candidate gene analysis revealed loci related to gravitropism, polar growth and hormonal signaling. No differences were observed for root biomass between lines carrying hap1 and hap2 for *qSRA-6A*, highlighting the opportunity to perform marker-assisted selection for the *qSRA-6A* locus and directly select for wide or narrow RSA, without influencing root biomass. Our study revealed that the genetic predisposition for deep rooting was best expressed under water-limitation, yet the root system displayed plasticity producing root growth in response to water availability in upper soil layers. We discuss the potential to deploy root architectural traits in cultivars to enhance yield stability in environments that experience limited rainfall.

## Introduction

Durum wheat (*Triticum durum* Desf.) is a major staple crop in the Mediterranean region (Shewry and Hey, 2015) and other semi-arid regions of the world (Araus et al., 2002). The crop is typically grown under rain-fed conditions where water scarcity is a major limiting factor for productivity, particularly when drought occurs during the flowering or grain filling period (Loss and Siddique, 1994; Belaid, 2000; Mohammadi et al., 2011; Bassi and Sanchez-Garcia, 2017). Due to climate change, rainfall patterns are predicted to change in most durum production regions worldwide, particularly in the Mediterranean region (Christensen et al., 2007; Carvalho et al., 2014). Therefore, breeding durum for water-limiting environments is a priority (Cattivelli et al., 2008; Boutraa, 2010).

Until recently, breeding programs have focused on above ground traits and direct selection for yield *per se*, while the crop’s “hidden-half,” i.e., the roots have been largely overlooked. Plant roots are important organs in determining grain productivity driven by water uptake and nutrient acquisition (Sharma et al., 2009; Ehdaie et al., 2012; Shen et al., 2013; Palta and Yang, 2014). Hence, improving RSA in breeding programs is a promising strategy to increase the resilience of durum wheat genotypes in drought-prone environments (Sanguineti et al., 2007; Manschadi et al., 2008). RSA has been recognized as one of the foundations for crop adaptation under water stress conditions (Manschadi et al., 2006; Christopher et al., 2008; Gregory et al., 2009; Asif and Kamran, 2011). Root length, density and root depth are the main components of RSA influencing water extraction in deep soils (King et al., 2003; Asif and Kamran, 2011; Carvalho et al., 2014). These adaptive features determining the root distribution in the soil profile have been associated with root growth angle (Nakamoto et al., 1991; Oyanagi et al., 1993; Oyanagi, 1994; Borrell et al., 2014). In durum wheat, seminal root angle (SRA) is representative of the mature RSA and provides a useful proxy because the trait can be easily phenotyped at the seedling stage (Tuberosa et al., 2002a,b, 2007; de Dorlodot et al., 2007; Fang et al., 2017; El Hassouni et al., 2018). For instance, a narrow SRA is associated with a higher proportion of roots at depth at the mature stage in wheat (Nakamoto and Oyanagi, 1994; Bengough et al., 2004; Manschadi et al., 2008), similar to root growth angle reported in other major crops like sorghum and rice (Omori and Mano, 2007; Uga et al., 2011; Mace et al., 2012). A narrow SRA can improve access to residual moisture in deep soils, particularly under terminal drought conditions (Manschadi et al., 2006; Reynolds et al., 2007; Christopher et al., 2008; Acuña and Wade, 2012; Hamada et al., 2012) and can prolong the grain filling period to improve yield (Blum et al., 1983; Lynch, 1995; Kashiwagi et al., 2005). On the other hand, wide SRA is associated with a shallow root system that may be beneficial for exploring the superficial soil layers and capturing in-season rainfall. Therefore, identifying the optimal RSA in each target environment is critical to guide breeding efforts (El Hassouni et al., 2018). Minor differences in the distribution of roots in the soil space can lead to major impacts on yield. For instance, results from modeling studies suggest that wheat yield would increase by 55 kg.ha^-1^ for each additional millimeter of water extracted from the soil during the critical grain filling stage (Manschadi et al., 2006; Kirkegaard et al., 2007; Christopher et al., 2013). Furthermore, a recent study examining RSA in durum wheat suggests that genotypes with deep root systems could increase grain yield up to 35% and thousand kernel weight by 9% in environments with limited moisture, compared to genotypes with shallow root systems (El Hassouni et al., 2018). The availability of large genetic variability in terms of rooting patterns and the high heritability of SRA (Manschadi et al., 2006, 2008; Maccaferri et al., 2016; Alahmad et al., 2018; El Hassouni et al., 2018) are two key factors suggesting that optimization of the roots could potentially deliver high yielding durum cultivars in water-limiting environments.

In comparison to aboveground traits, studying root traits have been a challenge for plant breeders (Zhang et al., 2009), largely due to lack of efficient and reliable root phenotyping methods and limited knowledge of the genetic control of root development (Tuberosa et al., 2002b; Zhang et al., 2009; Richards et al., 2010; Mace et al., 2012; Shen et al., 2013; Carvalho et al., 2014; Wang et al., 2014). Recently, a high-throughput, affordable and scalable phenotyping method for screening seminal root angle under controlled conditions has been developed, known as the ‘clear pot’ method (Richard et al., 2015), and has been successfully applied to durum wheat, barley, and bread wheat. The technique has facilitated direct phenotypic selection of SRA (Alahmad et al., 2018; Richard et al., 2018), phenotyping cultivars and breeding lines to investigate yield trends (El Hassouni et al., 2018; Robinson et al., 2018), and phenotyping of mapping populations required for QTL discovery (Robinson et al., 2016). While evaluation of mature RSA in the field is challenging, moderately efficient techniques have been developed, such as ‘shovelomics’ (Trachsel et al., 2011), soil coring (Wasson et al., 2014) and the ‘pasta strainer’ method (El Hassouni et al., 2018). Despite the challenges, good progress has been made to identify some of the genomic regions influencing RSA in durum wheat, with several bi-parental and association mapping studies published to date (Sanguineti et al., 2007; Cane et al., 2014; Maccaferri et al., 2016). A recent prioritization analysis of QTL detected in bi-parental and association mapping studies identified nine main QTL clusters on chromosomes 2A, 2B, 4B, 6A, 7A, and 7B, which appear to be most valuable for breeding applications (Maccaferri et al., 2016). However, further research is required to dissect the genetics of RSA in durum wheat that is relevant to breeders, along with the discovery of large effect QTL that are most desirable for marker-assisted breeding.

This study applied the ‘clear pot’ method to phenotype elite durum populations derived from crosses between Australian and ICARDA germplasm pools and performed a genome-wide association study (GWAS) using DArT-seq markers. A major QTL was identified on chromosome 6A that modulates growth angle, but not root biomass. This major QTL could be exploited and combined with root biomass, thus facilitating the development of new varieties with designer root systems that optimize resource capture in the soil profile targeting different environments.

## Materials and Methods

### Plant Material

A panel of 14 genotypes (Table 1) was evaluated for SRA under controlled conditions and nodal root angle in the field to investigate correlation between these traits. This included eight genotypes imported into Australia in 2015 from ICARDA’s durum wheat breeding program in Morocco (Fastoz2, Fastoz3, Fastoz6, Fastoz7, Fastoz8, Fastoz10, Outrob4, and Fadda98). The lines were preselected for drought adaptation and used as parents in breeding programs targeting marginal rainfall regions of West Asia and North Africa. Three Australian durum commercial varieties were also included (DBA Aurora, Jandaroi, Yawa) which are preferred by growers and the pasta industry due to high yield potential and protein content. In addition, bread wheat varieties Mace, Wylie and Scout were included with Mace and Scout used as standards of known root angle phenotype (Table 1).

**Table 1:** Details for the panel of 14 durum wheat and bread wheat standards examined in this study.

Genotype ID	Ploidy	Origin	Pedigree
DBA-Aurora	Tetraploid	Australia	Tamaroi^∗^2/Kalka//RH920318/Kalka///Kalka^∗^2/Tamaroi
Jandaroi	Tetraploid	Australia	110780/111587
Yawa	Tetraploid	Australia	Westonia/Kalka//Kalka/Tamaroi///RAC875/Kalka//Tamaroi
Outrob4	Tetraploid	ICARDA	Ouassel1/4/GdoVZ512/Cit//Ruff/Fg/3/Pin/Gre//Trob
Fadda98	Tetraploid	ICARDA	Awl2/Bit
Fastoz2	Tetraploid	ICARDA	T.polonicumTurkeyIG45272/6/ICAMORTA0463/5/Mra1/4/Aus1/3/Scar/GdoVZ579//Bit
Fastoz3	Tetraploid	ICARDA	Msbl1//Awl2/Bit/3/T.dicoccoidesSYRIG117887
Fastoz6	Tetraploid	ICARDA	Azeghar1/6/Zna1/5/Awl1/4/Ruff//Jo/Cr/3/F9.3/7/Azeghar1//Msbl1/Quarmal
Fastoz7	Tetraploid	ICARDA	CandocrossH25/Ysf1//CM829/CandocrossH25
Fastoz8	Tetraploid	ICARDA	MorlF38//Bcrch1/Kund1149/3/Bicrederaa1/Miki
Fastoz10	Tetraploid	ICARDA	Younes/TdicoAlpCol//Korifla
Mace	Hexaploid	Australia	Wyalkatchem/Stylet//Wyalkatchem[3798]
Scout	Hexaploid	Australia	Sunstate/QH-71-6//Yitpi[4113][4174][4177]
Wylie	Hexaploid	Australia	QT-2327/Cook//QT-2804[3596][3784]


**FIGURE 1:**
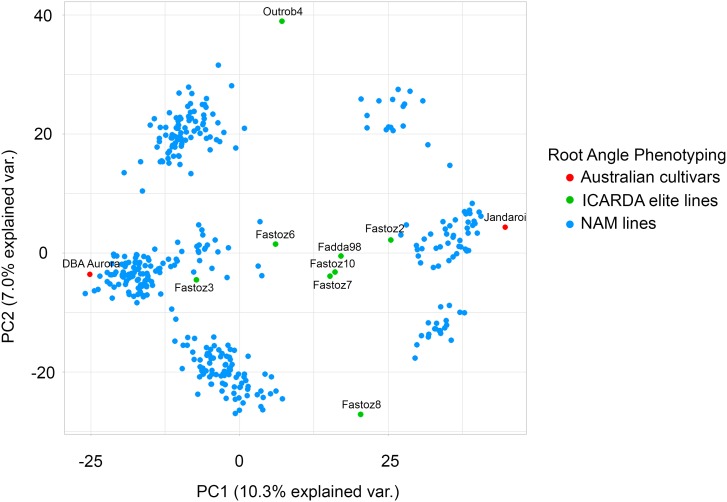
Population structure for the durum NAM lines evaluated for seminal root angle using the clear pot method. A principal component analysis based on pairwise modified Roger’s distances calculated from 2,541 polymorphic DArTseq markers was performed for the 393 NAM lines. The ten families NAM lines are derived from two Australian reference varieties (red) and ICARDA elite lines (green).

A subset of 393 durum recombinant inbred lines from a nested association mapping (NAM) population were evaluated for SRA and used for GWAS. The NAM population was generated by crossing the eight ICARDA lines listed above as ‘founders’ to the ‘reference’ Australian durum varieties Jandaroi and DBA Aurora. The speed breeding facility at The University of Queensland was used to rapidly progress through six generations of spring durum wheat in a year (Ghosh et al., 2018; Watson et al., 2018). The NAM resource comprises 10 donor × reference sub-populations of 92 F_6_ lines each (Figure 1). The subset of 393 lines evaluated for SRA was selected from the ten families based on agronomic appearance in the field.

### Phenotyping Seminal Root Angle Under Controlled Conditions

The panel of 14 genotypes including NAM parents and bread wheat standards (Table 1) were phenotyped for SRA, using the ‘clear pot’ method which is suitable for screening small grain crops (Richard et al., 2015; Robinson et al., 2016; Alahmad et al., 2018). In this experiment, clear pots were filled with composted fine, black-colored pine bark, consisting of 70% particles 0–5 mm in size, pre-mixed with 30% coco peat to increase the water-holding capacity. A randomized complete block design (RCBD) was adopted using *R V3.4.3* (R Core Team, 2017), with 15 replicates per genotype and 24 positions per 4 L pot. Pots were placed on the bench in a distinct column/row grid according to the RCBD design. Seeds were planted in the pots carefully positioning the embryo facing the wall of the pot and vertically with the radical pointed downward. This allows enhanced visibility of the seminal roots following germination. Plants were grown in the glasshouse under diurnal natural light conditions and constant temperature (17 ± 2°C) as recommended by Richard et al. (2015). Images were captured 5 days after sowing (seminal roots 3–5 cm in length) using a Canon PowerShot SX600 HS 16MP Ultra–Zoom Digital camera. The angle between the first pair of seminal roots was measured from the images using *ImageJ* software^1^. Two bread wheat genotypes were tested as standards, including Mace for wide and Scout for narrow SRA (Alahmad et al., 2018). The subset of 393 NAM lines, parents and standards were subsequently phenotyped for SRA using the same procedure as described above.

### Phenotyping Nodal Root Angle in the Field

The panel of 14 genotypes was evaluated for nodal root angle in the field using a ‘shovelomics’ approach (Trachsel et al., 2011). The field experiment was conducted at The University of Queensland Gatton Research Station (27°32′45″ S; 152°19′44″ E) known for summer dominant rainfall and clay soils, Queensland, Australia, in 2017. RCBD was adopted using *R V3.4.3* (R Core Team, 2017) where each genotype was replicated 3 times in 7.5 m^2^ yield plots (5 rows spaced 0.3 m × 5 m long). Once all genotypes had reached anthesis, 10 plants per genotype were randomly selected and excavated from the internal two rows. Plants were manually removed to a depth of 20 cm. Excavated plants were then vigorously shaken to remove the loose dry soil before images of the crown roots were captured using a smart-phone camera in the field. These images were then analyzed and the outer angle of the nodal roots was measured using *Image J* software.

### Analysis of Phenotype Data

All phenotypic data analyses were performed in *R V3.4.3* (R Core Team, 2017). The root growth angle phenotypes for parental lines (*n* = 14) in the glasshouse and the field, as well as the subset of NAM lines (*n* = 393 F_6_ lines) in the glasshouse, were measured using ImageJ (Schneider et al., 2012). Statistical analyses of the root growth angle measurements were performed using *ASReml-R* (Butler et al., 2009). Best linear unbiased estimates (BLUEs) were calculated for each individual, including the parental lines and the NAM lines, based on root angle data generated in the glasshouse experiment using the ‘clear pot’ method. To account for spatial variation a mixed linear model was fitted. In this model, the genotypes were fitted as fixed effect, while the replicate, pot and position were fitted as random terms. The field experiment of the parental lines was conducted to investigate the correlation of mature root growth angle under field conditions with measurements of roots from plants grown under glasshouse conditions at early growth stage. BLUEs for the field data were obtained by fitting a linear mixed model with genotype as a fixed effect and the plot coordinates (row and column) as random effects. The BLUEs for the subset of NAM lines were used as phenotypes in the GWAS analyses. Significance of differences in root biomass between genotypes and between SRA haplotypes were tested using Tukey’s test for general linear hypothesis testing based on the linear models described above. Data derived from image analysis of rhizoboxes, and the anatomical traits from the cross sections were also analyzed and differences between the means of genotypes were tested for significance based on a Fisher’s Least Significant Difference (LSD) test for multiple comparison with a family-wise error rate 5%.

### Genotyping and Curation of Marker Data

All 10 families of the durum NAM population were genotyped using the Diversity Arrays Technology (DArT) genotyping-by-sequencing platform (DArTseq; Figure 1). Leaf tissues were sampled from F_6_ plants and genomic DNA was extracted according to the protocol provided by DArT. Genotyping resulted in a total of 13,395 DArTseq markers which were ordered according to their genetic positions in the consensus map (version 4.0), provided by Diversity Arrays Technology Pty Ltd., Canberra, Australia. Markers with a frequency of heterozygotes of ≥0.1 and missing calls of ≥20% were omitted. Markers with ≥30% missing data and a minor allele frequency of <3% were omitted and only genotypes with ≤20% missing marker information were considered, resulting in a selection of 2,541 high-quality, polymorphic DArTseq markers in 393 durum wheat lines which were used for the subsequent genetic analyses.

### Genome-Wide Association Mapping

The 2,541 high-quality genome-wide markers were used to investigate marker-trait-associations (MTA) for SRA. Significances for MTAs were calculated in a two-step mixed linear model approach that increases detection power without increasing the empirical type I error (Stich, 2009). We used a mixed model implemented in the R package *GenABEL* (Aulchenko et al., 2007) which adjusted for population stratification by including identity-by-state estimates for genotype pairs (as a kinship matrix) and a principal component adjustment that uses the first four principal components as fixed covariates. To reduce the type I error rate, we applied a stringent Bonferroni cut-off threshold of –log_10_(*p*-value) = 4.67 (α = 0.05) for SRA (Bland and Altman, 1995). The major SRA QTL exceeding this threshold was then compared with previously identified drought-related and yield component QTL in an alignment approach.

Local LD of the significant markers on chromosome 6A for SRA was calculated and used to group markers into one QTL. Markers with pairwise *r*^2^ values > 0.60 were assigned to an LD block and included in the haplotype analysis, resulting in eight haplotype variants which were observed in the population. Haplotype networks, showing TCS genealogies between haplotype variants (Clement et al., 2000), were calculated using *PopART*^2^ (Leigh and Bryant, 2015). The network nodes were colored according to the average SRA in the respective haplotype groups. To investigate the effect of the SRA QTL on the growth angle measurements while correcting for variability due to genetic background, we selected three sub-NAM populations, segregating for the SRA allele combinations associated with narrow and wide SRA including DBA Aurora × Outrob4, DBA Aurora × Fastoz8, DBA Aurora × Fastoz3. We compared the mean SRA of lines that carried the two most frequent haplotypes hap1 and hap2 within the families separately. A Tukey’s test was performed to test phenotypic differences in SRA between the haplotype groups within each family. The haplotype effects on root angle phenotypes, were visualized using *GraphPad Prism V6* (Graphpad Software Inc.).

### Evaluating Root and Shoot Biomass Effects of Root Angle QTL

To investigate whether the identified major SRA QTL is also associated with pleiotropic differences in root or shoot biomass, a glasshouse experiment was conducted under controlled conditions. A total of 40 closely related genotypes segregating for SRA QTL were evaluated, including 20 lines carrying hap1 and 20 lines carrying hap2. The panel was phenotyped for root biomass using the method reported by Voss-Fels et al., 2017 with some modifications. Here, ANOVA pots (ANOVApot^®^, 137 mm diameter, 140 mm height) were filled with 1,650 g of sand (with particle size ranging from 0.075–4.75 mm) to facilitate efficient root washing. An RCBD was used for the experimental design, with four plants per genotype in each 1.4 L pot, in three replicates. Fifteen pots were placed in a container fitted with capillary mats to provide sufficient water and nutrient supply. A hydroponic solution was added to each container (1.50 mL of Cultiplex per L of deionized water) and was maintained at the same level over the course of the experiment. The concentration of the nutrient solution was gradually increased as the plants developed and required additional nutrient supply (days 1–10: 1.50 mL/L, days 11–17: 2 mL/L, days 18–22: 2.50 mL/L, days 23–26: 3 mL/L).

Seeds were germinated using a cold treatment (4°C) for 3 days to promote synchronous germination. The germinated seeds were transplanted to the sand-filled plastic pots and grown under diurnal (12 h) photoperiod in a temperature-controlled glasshouse (22/17°C; day/night). At 26 days after sowing (early tillering stage) plants were extracted with minimum disruption to the roots by placing the pot in a water-filled container and carefully washing off the remaining sand in clean water. The roots and shoots from each pot were separated and placed in a dehydrator at 65°C for 72 h before dry weight was measured.

### Evaluation of Root Ideotypes Under Well-Watered and Drought Conditions

To investigate the potential for breeding cultivars with different root ideotypes, durum NAM lines representative of four distinct root ideotypes (root angle-root biomass; wide-low, wide-high, narrow-high, and narrow-low) were evaluated using rhizoboxes, similar to those described by Singh et al. (2010). Representative lines were selected based on extreme root angle and biomass phenotypes, as well as haplotype information for the major SRA QTL. Briefly, germinated seeds were sown in rhizoboxes (4 cm × 26 cm × 60 cm) at a depth of 3 cm and maintained under diurnal photoperiod (12 h) and a temperature of 22/17°C (day/night). An RCBD design was adopted in three replicates as blocks in two treatments (well-watered and drought). Four plants per ideotype were planted in each rhizobox. Four rhizoboxes were placed in a container filled with 300 mL water to supply plants with water from the bottom of the rhizoboxes in both treatments. Following sowing, all chambers were watered daily until 1 week after sowing. The well-watered (control) treatment received daily watering from the top of the rhizobox while the drought treatment received no additional water and was subjected to severe water-limitation in the upper layer of the soil. The percentage of soil moisture was measured weekly over the course of the experiment using a soil moisture meter (PMS-714; Lutron Electronic; probe length 22 cm and probe diameter 1 cm) at a depth of 50 cm. Images of the rhizoboxes were captured after 5 weeks and analyzed using *GIA Roots* software (Galkovskyi et al., 2012). The images were cropped into three equal sections at 0–20, 20–40, and 40–60 cm to evaluate root distribution at various soil depths.

To investigate differences in root anatomy associated with the root angle QTL or root ideotype, the stele diameter (SD) and metaxylem area (MXA) was measured for root tissue sampled 10 cm from the seminal root apex in both well-watered and drought treatments. Roots were hand sectioned with a razor blade using a dissecting microscope. The sections were stained with Toluidine Blue O. Images of the root sections were processed using a Zeiss Axio Microscope (Scope.A1 with 100× magnification). All image analyses were processed using *ZEN lite 2012 software* (blue edition, Jena, Germany).

### Alignment of Previously Reported QTL for Root and Yield Component Traits

The QTL reported in this study was positioned on the Svevo durum physical map (Maccaferri et al., 2019). The previously reported QTL associated with RSA, distribution and growth angle (Maccaferri et al., 2016) were also projected onto the map using *MapChart V2.3* (Voorrips, 2002). In addition, the previously reported QTL associated with the yield components (TKW, grain yield per spike) and the quality parameter yellow pigment concentration were aligned on the chromosomal region of interest (Golabadi et al., 2011; Roncallo et al., 2012; Maccaferri et al., 2016; Mengistu et al., 2016).

### Candidate Gene Analysis

#### Mapping of Marker Genes in the Bread Wheat Reference Genome IWGSC RefSeq v.1.0

Identified peak markers were mapped onto the homologous bread wheat pseudochromosome 6A using the recently published RefSeq v1.0 annotations (Appels et al., 2018). High confidence (HC) and low confidence (LC) RefSeq v1.0 gene models were extracted from the identified region and used in further analyses. Similarity searches were carried out using BLASTn with high stringency settings (with an *e*-value cut-off of 1e-100). Collinearity analysis of Chromosome 6AL between *T. durum* and *Triticum aestivum* regions were performed using Pretzel^3^. Mapped markers and genes expressed in root tissues in seedling stage were used for the analysis.

#### Gene Expression Analysis and Functional Predictions

Gene expression patterns of the selected bread wheat gene homologs on chromosome 6A were analyzed using the developmental gene expression atlas of polyploid wheat (Ramírez-González et al., 2018); Wheat eFP Browser at http://bar.utoronto.ca/efp_wheat/cgi-bin/efpWeb.cgi and visualized in R using the *Morpheus* package^4^.

Translated sequences of selected durum gene models were subjected to functional KEGG pathway analysis using blastKOALA (Kanehisa et al., 2016). Potential interacting proteins were analyzed in STRING (Szklarczyk et al., 2015) using the reference genomes of *Brachypodium distachyon*, *Hordeum vulgare*, *Oryza sativa*, *Zea mays*, and *Arabidopsis thaliana* as data background.

## Results

### Variation for Root Angle: From Glasshouse to Field

**FIGURE 2:**
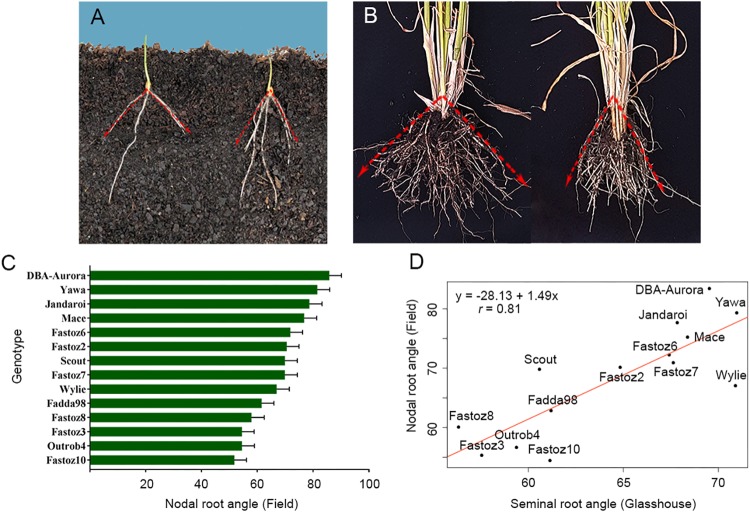
Root growth angle phenotypes measured in important durum wheat cultivars from Australia and ICARDA: **(A)** seminal root angle for Australian variety DBA Aurora (left, wide root angle) and an ICARDA elite founder line Outrob4 (right, narrow root angle) screened using the clear pot method, and **(B)** in the field using shovelomics method. **(C)** Nodal Root growth angle field measurements of 14 parental lines used for NAM population development. Correlation between seminal root angle in the glasshouse and mature roots in the field, *r* = 0.81, *P* = 0.00038 **(D)**.

In this study, a panel consisting of the parents of the NAM population and standard lines with previously analyzed root characteristics was evaluated for SRA under controlled conditions in the glasshouse (Figure 2A) and nodal root angle under field conditions (Figure 2B). Phenotypes displayed by standards were as expected under glasshouse and field conditions, however, less variation under field conditions was observed. For example, the SRA for standard lines under glasshouse conditions were 110.1° (Mace) and 62.6° (Scout) compared to 76.8° (Mace) and 69.9° (Scout) for nodal roots under field conditions (Figure 2C). Although the absolute values varied between glasshouse and the field, Mace consistently displayed a wider root angle than Scout across both experiments.

In both experiments, the ICARDA founder lines generally displayed a narrow root growth angle in comparison to the Australian durum cultivars. For example, the SRA for ICARDA founder lines ranged from 50.2–60.7° under glasshouse conditions and 51.8–61.6° in the field. Australian cultivars ranged from 83.0–97.8° under glasshouse conditions and 78.7–85.8° in the field. A strong correlation between seminal root angle in the glasshouse and mature root angle in the field was observed as shown in the regression analysis in Figure 2 (*r* = 0.81, *P* = 0.00038), wherein the panel of 14 lines showed consistent root growth angle phenotypes (Figure 2D).

### Segregation for Root Angle in the NAM Lines

**FIGURE 3:**
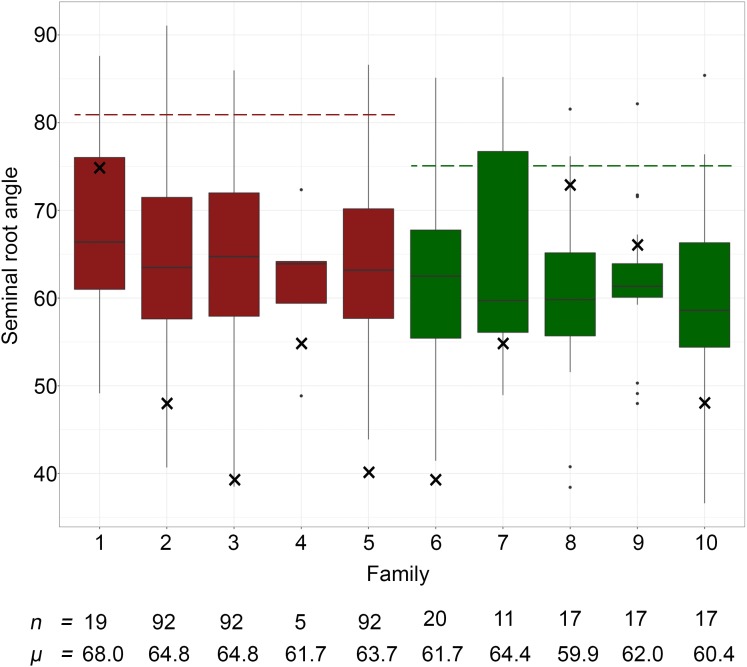
Seminal root growth angle measurements of the 10 NAM families. Families 1 to 5 (red) share DBA Aurora as the common parent, and families 6–10 (green) share Jandaroi as the common parent. Family 1 = DBA Aurora × Fastoz7; Family 2 = DBA Aurora × Outrob4; Family 3 = DBA Aurora × Fastoz8; Family 4 = DBA Aurora × Fadda98; Family 5 = DBA Aurora × Fastoz3, Family 6 = Jandaroi × Fastoz8; Family 7 = Jandaroi × Fastoz10; Family 8 = Jandaroi × Fastoz6; Family 9 = Jandaroi × Fastoz2; Family 10 = Jandaroi × Outrob4. Boxplots display the quartile range and median SRA (horizontal line) of individuals within each of the 10 sub-NAM populations. The broken red line displays the mean SRA value of DBA Aurora and the broken green line displays the mean SRA value of Jandaroi; × represents the mean SRA value of ICARDA founder lines; *n* represents the number of individuals in each family; *μ* represents the mean SRA value of each family.

A high degree of variation for SRA was observed among the 393 NAM lines, with adjusted means ranging from 36.6–91.1° (Figure 3). In families derived from DBA Aurora (SRA = 81.1°), the SRA ranged from 38.5–91.1° and in the families derived from Jandaroi (SRA = 75.5°), the SRA ranged from 36.6–85.4°. In particular, three families (Family 2, Family 3 and Family 5) derived from crosses between DBA Aurora (widest root angle) and three ICARDA founder lines with the narrowest root angle (Outrob4 = 48.7°, Fastoz8 = 39.7° and Fastoz3° = 41.8°, respectively) displayed little transgressive segregation, with a number of lines showing slightly narrower or wider SRA phenotypes than the respective parents. For example, SRA of the individuals ranged from 40.7–91.1°, 38.5–86.0°, and 43.9–86.6° for Families 2, 3, and 5, respectively. Family 1 displayed a higher degree of transgressive segregation. In addition, two families (Jandaroi × Fastoz8 and Jandaroi × Outrob4, i.e., families 6 and 10, respectively) also displayed transgressive segregation, ranging from 41.5–85.1° (Family 6) and 36.6–85.4° (Family 10).

### A Major QTL for Root Growth Angle Is Located on Chromosome 6A

**FIGURE 4:**
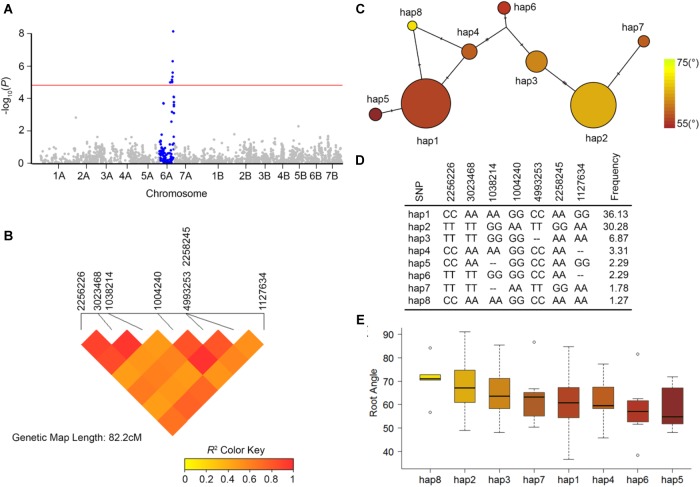
Genome-wide association mapping for seminal root angle in 393 durum lines using 2,541 high quality DArTseq markers (minor allele frequency > 5%). **(A)** Manhattan plot showing chromosome 6A (blue) with significant marker-trait association at Bonferroni significant threshold 4.67 (red horizontal line). The *x*-axis displays the DArTseq markers on 14 chromosomes; y-axis is the –log_10_(*P*). **(B)** Heat map showing pairwise linkage disequilibrium (LD) between 7 significant markers representing major seminal root angle QTL on chromosome 6A (*qSRA-6A*). Color gradient represents LD as *r*^2^. **(C)** Haplotype network of 8 haplotype variants of the *qSRA-6A* that were found in the 393 NAM lines. Size of the circles represents the frequency of each haplotype in the population. Node color indicates mean seminal root angle for lines carrying the haplotype. **(D)** Allelic marker-combination of the 8 haplotypes for the 7 DArTseq markers and the frequency value of each haplotype. **(E)** Seminal root angle variation in each haplogroup.

A total of seven highly significant markers for SRA were detected on chromosome 6A [at Bonferroni threshold = –log_10_(*P*) 4.67; Figure 4A]. A single major QTL region was defined based on high LD (*r*^2^ > 0.60) between pairwise markers, resulting in a QTL interval defined by the outer flanking markers 2256226 (86.46 cM DArTseq V4 consensus map) and 1127634 (94.68 cM DArTseq V4 consensus map) (Figure 4B). For this QTL, eight main haplotypes were detected (Figure 4C). Hap1 and hap2 were the most frequent allelic variants in the subset of NAM lines (frequency = 36.1 and 30.3%, respectively) (Figure 4D). The mean SRA for genotypes in the eight defined haplotype groups ranged from 57.8–71.0° (Figure 4E). Comparison of SRA between the most frequent haplotypes hap1 and hap2 revealed a highly significant difference of 7.7° (*SE* = 1.2, *P* = <0.001) across all families segregating for the QTL in both genetic reference backgrounds DBA Aurora and Jandaroi.

**FIGURE 5:**
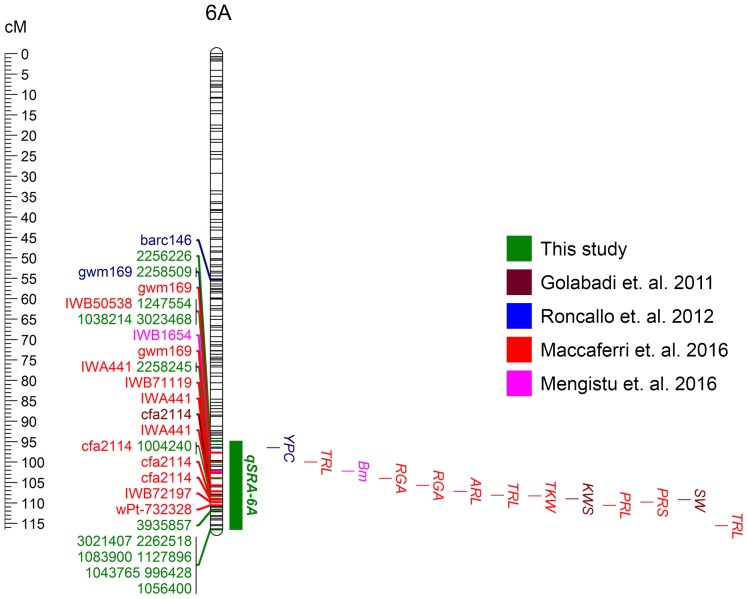
Major QTL for seminal root angle (*qSRA-6A*) positioned on the Svevo durum physical map (Mbp), along with QTL reported in previous mapping studies including root system architecture traits (TRL, total root length; RGA, root growth angle; ARL, average root length; TRL, total root length; PRL, primary root length; PRS, primary root surface), yield component traits (Bm, biomass; TKW, thousand kernel weight; KWS, grain yield per spike; SW, spike dry matter) and a quality trait (YPC, yellow pigment concentration).

The QTL detected in this study and previously reported QTL in the same chromosomal region (Golabadi et al., 2011; Roncallo et al., 2012; Mengistu et al., 2016; Maccaferri et al., 2016) were positioned onto the durum reference genome (Svevo physical map, Maccaferri et al., 2019; Figure 5). The major QTL found in our study (*qSRA-6A*) was found to be co-located with previously reported durum QTL for root growth angle, total root length and root biomass, as well as QTL for yield components and quality traits (Figure 5).

### *qSRA-6A* Influences Root Angle but Not Root Biomass in Different Genetic Backgrounds

**FIGURE 6:**
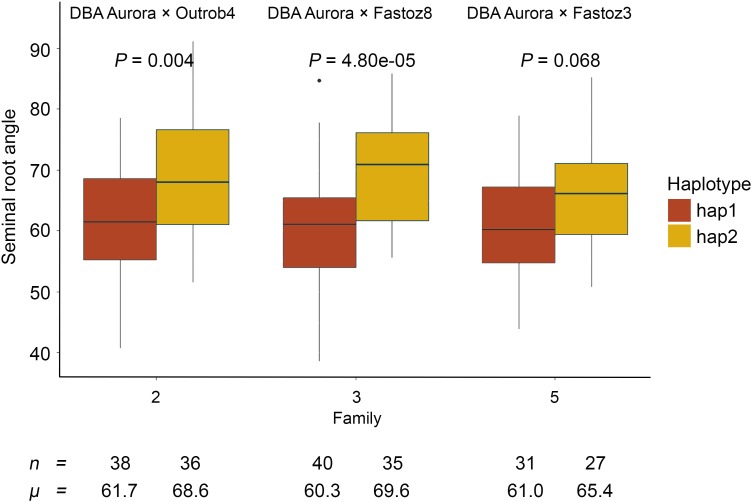
Seminal root angle measurements in three NAM families segregating for the most common haplotype of the QTL *qSRA-6A*. Families were derived from crosses between DBA Aurora to three ICARDA lines (Outrob4, Fastoz3, and Fastoz8). In each family, mean SRA value of individuals carrying hap1 and hap2 was compared. The colors represent the two haplotype groups, *n* represents the number of individuals carrying different haplotype groups, *μ* represents the mean SRA value of each haplotype group, and *P* represents significance from an Honestly Significant Difference (HSD) test for the difference between the two haplotype groups within each family.

To evaluate haplotype effects for *qSRA-6A*, we compared the most common haplotypes in three families that were segregating for the QTL. The three families derived from crossing Outrob4, Fastoz8 and Fastoz3 with the common reference parent DBA Aurora were tested as these families segregated for hap1 and hap2 of the major QTL (Figure 6). The phenotypic differences in SRA between individuals carrying hap1 and hap2 were significant for family 2 and 3 and followed a similar trend in family 5. Amongst the families, the difference in SRA for lines carrying hap1 versus hap2 ranged between 4.4–9.3°. The largest effect was evident in the DBA Aurora × Fastoz8 family (60.3 and 69.6° for hap1 and hap2, respectively).

To investigate if the contrasting main haplotypes for *qSRA-6A* were only associated with root architectural differences or with overall plant development we conducted a subsequent experiment in which we assayed root and shoot biomass for a subset of 40 genotypes that represented hap1 (*n* = 20) and hap2 (*n* = 20). Comparing dried total root biomass, total shoot biomass and the root/shoot ratio of this subset showed no significant differences between the two main haplotype groups. Average values for hap1 and hap2 were 0.641 g/line and 0.638 g/line for total root biomass, 1.007 g/line and 1.023 g/line for total shoot biomass and 0.643 and 0.631 for root/shoot ratio.

### Root Distribution and Anatomy of Four Root System Ideotypes

**FIGURE 7:**
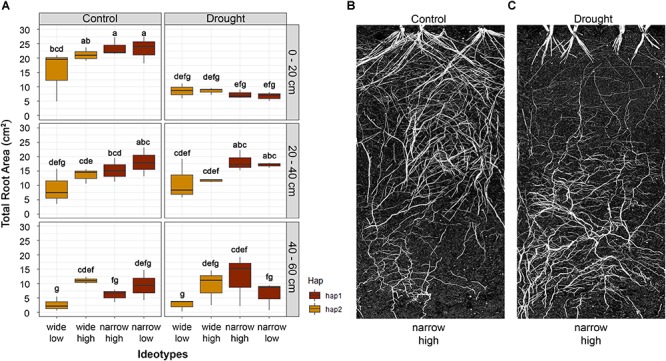
Root area distribution of the four root ideotypes wide-low, wide-high, narrow-high and narrow-low at different depths of the growth chamber. **(A)** Boxplots display root distribution of the four ideotypes under control (well-watered) and drought conditions. The colors represent the two haplotype groups of the root angle *qSRA-6A.* Letters above boxplots indicate significance difference between the four root ideotypes using least significant difference (LSD) test at α = 0.05. Visualization of a narrow-high root ideotype under **(B)** controlled (well-watered) and **(C)** drought conditions is shown.

Root distribution under well-watered and drought conditions was investigated for four root system ideotypes in rhizoboxes (Supplementary Figure S1). Soil moisture of the rhizoboxes decreased dramatically with significant differences between treatments from 3 weeks after sowing (Supplementary Figure S2). Overall, plants in the drought treatment had less total root area (area of the roots in the images) (Figure 7A) and significantly reduced crown root growth (Figure 7B,C). Unexpectedly, the lines carrying the narrow allele were responsive to localized water availability in the upper strata (Figure 7B), while in the drought treatment root proliferation shifted deeper into the strata in response to soil moisture at depth (Figure 7C). For example, root ideotype ‘narrow-high’ produced significantly higher root area (23.54 cm^2^) in comparison to wide ideotypes (*P* < 0.05, wide-high = 21.15 cm^2^; wide-low = 15.11 cm^2^) in the upper soil layer (0–20 cm) of the rhizobox under well-watered conditions. In addition, the ‘narrow-high’ ideotype produced a significantly higher root area distribution under drought conditions in the middle and deepest soil layers (20–40 cm = 18.15 cm^2^; 40–60 cm = 12.13 cm^2^) when compared to both wide ideotypes (20–40 cm; 10.95–11.60 cm^2^, *P* < 0.05–0.1 and 40–60 cm; 2.60–9.35 cm^2^, *P* < 0.01–0.4; Figure 7).

**FIGURE 8:**
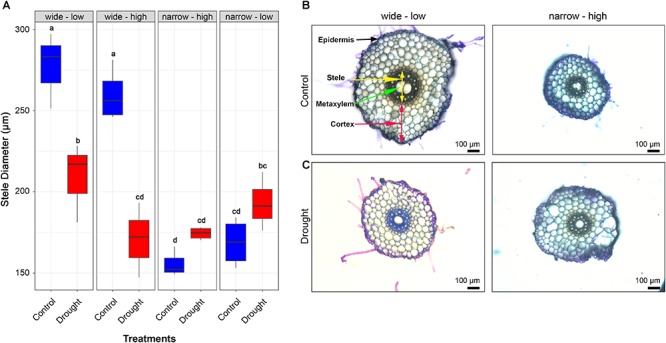
Anatomical features of roots sampled from durum wheat genotypes representative of distinct root system ideotypes. **(A)** Stele diameter of the four root ideotypes of samples collected 10 cm from root apex, under well-watered (control; boxplot colored in blue) and drought conditions (boxplot colored in red). Mean stele diameter with different letters above the boxplot are significantly different. Radial root cross sections on seminal root at 10 cm from root apex displaying anatomical variation in the root ideotype wide root angle with low biomass **(B)** and narrow root angle with high root biomass **(C)** under well-watered and drought conditions, scale bars in the cross sections = 100 μm.

To investigate associations between root architecture and root anatomical features, stele diameter (SD) and metaxylem area (MXA) were measured for the four ideotypes in the rhizobox experiment. Results suggested a strong link between root angle QTL *qSRA-6A* and SD (Figure 8A,B), as well as MXA at depth under well-watered conditions. Under well-watered conditions, the mean MXA for wide-low and wide-high root ideotypes were 11,930 ± 2,100 μm^2^ and 11,781 ± 3,287 μm^2^, respectively, in comparison to 3,137 ± 353 μm^2^ and 3,821 ± 794 μm^2^ for the narrow-high and narrow-low, respectively. However, the link was not evident under drought conditions. Under drought conditions, the mean MXA for wide-low and wide-high root ideotypes were 6,627 ± 1,203 μm^2^ and 4,543 ± 1,076 μm^2^, respectively, in comparison to 3,986 ± 256 μm^2^ and 5,577 ± 765 μm^2^ for the narrow-high and narrow-low, respectively (Figure 8C). Overall, wide root angle genotypes showed significantly reduced SD and MXA under drought conditions (*P* < 0.05). In addition, the ‘narrow-high’ ideotype which displayed the highest proportion of roots at depth, also showed smaller MXA under drought conditions at depth.

### Candidate Genes Underpinning the 6A QTL

Markers that were found to be significantly associated with SRA mapped to the distal end of chromosome 6A in durum and bread wheat. The length of the marked region was 22.82 Mbp in durum and 22.81 Mbp in bread wheat. These regions contain 393 gene models in durum, while 515 HC and 34 LC gene models were identified in bread wheat. The homologous genes had a high level of collinearity between the terminal regions of chromosome 6A in the *T. durum* reference cultivar Svevo and the bread wheat reference genome RefSeq v1.0 (Supplementary Figure S3).

Gene expression patterns were analyzed using the high-resolution tissue and stage-specific RNAseq data of Azurhnaya spring wheat (Winter et al., 2007; Ramírez-González et al., 2018). Altogether 206 genes show root specific expression during the plant life cycle, from which 76 genes show significant expression during early root development stages (radicle and roots at the seedling stage, one leaf and three leaf stage roots and root apical meristem tissues; Supplementary Figure S3).

**FIGURE 9:**
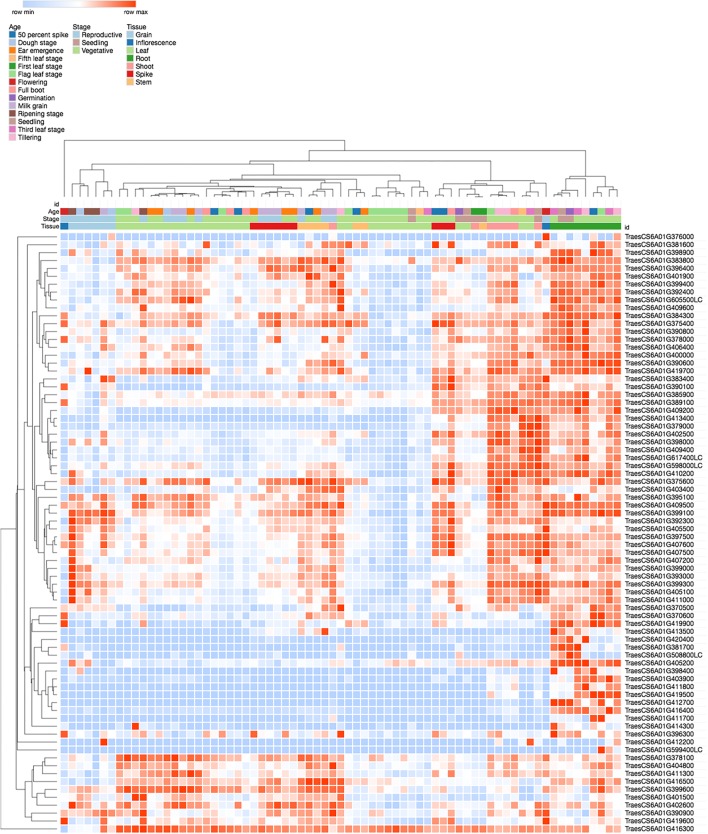
Expression patterns of bread wheat homologs at different age, stage, and tissue specific RNAseq libraries. Heatmap (blue = low; red = high) displayed high expression in root tissues (green) during seedling (pink) and vegetative stage (light green). Genes primarily expressed in the root tissues at seedling and vegetative stages are listed in Table 2.

Transcript expression patterns from various tissues both at seedling stage, vegetative and reproductive stages are represented in Figure 9. Of these, 15 genes were primarily enriched in the root tissues during early root development (Figure 9 and Table 2).

**Table 2:** List of 15 candidate genes identified using the homologous chromosome 6A genomic region of bread wheat through functional analysis of *qSRA-6A.*

Gene ID	Homologous RefSeq v1.0 Gene ID	Molecular function	KEGG pathway
TRITD6Av1G217760	TraesCS6A01G381700	Cinnamoyl CoA reductase	Monolignol biosynthesis
TRITD6Av1G218270	TraesCS6A01G383800	Glutathione cytosolic	Glutathione metabolism
TRITD6Av1G218390	TraesCS6A01G384300	desumoylating isopeptidase 1-like	
TRITD6Av1G220070	TraesCS6A01G392400	3-ketoacyl- thiolase peroxisomal	Fatty acid metabolism, Jasmonic acid biosynthesis, Beta-oxidation
TRITD6Av1G221500	TraesCS6A01G396400	Serine/threonine-protein phosphatase	Translation/mRNA surveillance pathway
TRITD6Av1G222970	TraesCS6A01G605500LC	Membrane protein	
TRITD6Av1G223490	TraesCS6A01G407600	F-box family protein	
TRITD6Av1G223520	TraesCS6A01G407600	F-box family protein	
TRITD6Av1G223760	TraesCS6A01G409500	Transmembrane protein	
TRITD6Av1G223780	TraesCS6A01G409600	Electron transfer flavoprotein beta-subunit	leucine catabolism and in phytol degradation
TRITD6Av1G224460	TraesCS6A01G412700	Protein kinase, Wall-associated receptor kinase 2	MAPK signaling
TRITD6Av1G224970	TraesCS6A01G413500	Ripening-related protein, RIPER1	
TRITD6Av1G225080	TraesCS6A01G508800LC	12-oxophytodienoate reductase 2	Fatty acid metabolism, Jasmonic acid biosynthesis
TRITD6Av1G225140	TraesCS6A01G414300	Disease resistance protein RPM1	Plant/pathogen interaction
TRITD6Av1G225520	TraesCS6A01G414300	Disease resistance protein RPM1	Plant/pathogen interaction


In the bread wheat genome (RefSeq v1.0 chr 6A region) the mapped markers overlap with gene models representing a NAC transcription factor (TraesCS6A1G386700), a fatty acid hydroxylase family protein (TraesCS6A1G384600), a PRONE protein (TraesCS6A1G405000) and a SAWADEE homeodomain protein 2 (TraesCS6A1G420700). The position of the peak marker from the SRA QTL *qSRA-6A* mapped to the exon region both in the NAC domain-containing protein (3023468) and the PRONE protein (3935857). The observed SNP caused 8:T > A and 7:C > G nucleotide changes, respectively, which resulted in an amino acid change to the translated protein.

Translated durum protein sequences mapped to the QTL region were subjected to KEGG pathway analysis. From the 387 *T. durum* sequences, 114 proteins had significant blast hits in the KEGG database. The following KEGG pathways were enriched: 13 proteins involved in pathogen defense mechanisms, seven proteins in secondary metabolite biosynthesis (monolignol biosynthesis) and five proteins in fatty acid metabolism [fatty acid biosynthesis, jasmonic acid (JA) biosynthesis and beta-oxidation]. Genes significantly expressed in the radicle and roots at the seedling stage as well as roots and root apical meristem at the three leaf stage were analyzed in more detail, using the STRING database to predict potential interacting protein networks. Studies using related monocot species (*B. distachyon*, *H. vulgare*, *O. sativa*, and *Z. may*s) as well as the model dicot species *A. thaliana* indicated the conserved patterns of interacting proteins enriched in functions involved in monolignol biosynthesis, fatty acid metabolism, jasmonic acid metabolism and beta-oxidation. Proteins belonging to plant–pathogen interaction pathways were also detected using both the moncot and dicot data backgrounds. However, homologous proteins of *Arabidopsis* were also related to fatty acid metabolism pathways. Extended interaction networks in all backgrounds also highlighted proteins that are identified in auxin metabolism.

## Discussion

### A Major QTL on 6A Determines Seminal Root Growth Angle

Here, we report a QTL on chromosome 6A (*qSRA-6A*) that has a significant effect on root growth angle in a subset of 393 durum NAM lines. The co-location of *qSRA-6A* with previously mapped QTL in durum wheat for various root traits, including root length, root surface and root biomass (Maccaferri et al., 2016; Mengistu et al., 2016) suggests that this chromosomal region has a major impact on root development. In addition, *qSRA-6A* also aligned with genomic regions influencing yield components and quality parameters, such as thousand kernel weight and yellow pigment content (Golabadi et al., 2011; Roncallo et al., 2012; Mengistu et al., 2016). Therefore, this region appears important for root system development, which may impact other agronomically important traits including grain yield and end-use quality parameters. Analysis of local LD around the main QTL peak showed high levels of pairwise LD between seven SRA-associated markers. Similar to reported observations for root traits in bread wheat (Voss-Fels et al., 2017) this suggests strong directional selection for this chromosomal block, resulting in a block-wise co-inheritance of markers in tight LD due to strong allelic fixation in important durum wheat germplasm (Tuberosa et al., 2002a; Hayes et al., 2007). Since root architecture directly affects the Source-sink relationship, it is likely that the underlying genetic mechanisms for drought-adaptive traits, such as root growth characteristics, also influence above ground traits like spike grain weight, TKW, spike dry weight and grain quality, which facilitates detection of similar QTL in segregating populations.

A recent study on SRA in bread wheat suggests that root angle is under complex genetic control with multiple small effect QTL involved (Richard et al., 2018). In barley, similar to our study, seminal root traits were reported as being affected by a major QTL on chromosome 5H (Robinson et al., 2016). In maize, a major QTL was reported as constitutive and was associated with root growth angle, root branching and root thickness. This QTL exhibited consistent strong effects under glasshouse and field conditions with different water treatments (Giuliani et al., 2005). In sorghum, four QTL for nodal root angle were mapped, two of which had a major effect and appeared to co-locate with previously identified QTL for stay-green expressed under low moisture conditions (Mace et al., 2012).

On the other hand, *VERNALIZATION1* (*VRN1*), which controls flowering time in cereals like wheat and barley, was found to modulate RSA in bread wheat and barley (Voss-Fels et al., 2018a). The QTL identified in this study provides the opportunity to introgress novel diversity into durum and bread wheat to modulate RSA, potentially leading to improved performance under specific environmental conditions.

### Promising Candidate Genes for *qSRA-6A* Have Root Growth-Related Functions

Analyses revealed that the position of the mapped markers of *qSRA-6A* overlaps with a genomic region enriched with genes related to gravitropism, polar growth and hormonal signaling. Notably, genes that are expressed only in root tissues at early stages of plant development (e.g., seedling and one-leaf stage) are involved in pathways such as fatty acid metabolism, jasmonic acid biosynthesis, and monolignol biosynthesis, that might be related to root angle variations in the analyzed phenotypes. Fatty acid metabolism and beta-oxidation play a significant role in the early germination steps when reserve lipids are mobilized to serve as respiratory substrates and to sustain the growth of the seedling. Among the identified early root development-related genes, genes encoding a 3-ketoacyl-CoA thiolase-like protein, Electron transfer flavoprotein beta-subunit and 12-oxophytodienoate reductase 2 and glutathione reductase are related to fatty acid metabolism and beta-oxidation in barley, rice, maize.

During triacylglycerol degradation, fatty acids are released and channeled into gluconeogenesis. Beta-oxidation is also essential to produce secondary metabolites in oxylipin signaling such as the 12-oxophytodienoic acid (OPR2) and jasmonic acid, which serve as signaling compounds in plant growth and pathogen defense mechanisms (Christine and Clifford, 2009; Wasternack and Hause, 2013). An inhibiting role of OPR2 on seed germination has been described (Dave et al., 2011; Dave and Graham, 2012), showing the interaction between 12-oxophytodienoic acid and abscisic acid that leads to increased ABA isensitive5 (ABI5) gene expression and suppressed germination. The identified durum OPR2 shows close homology to OPR5 in maize and OPR2 in barley; both of which are known to be involved in Jasmonic-acid biosynthesis pathways (Helmut et al., 2000). In roots, the growth inhibition by JA and OPR2 occurs via cross-talk with auxin and possibly other hormones, such as gibberelic acid (GA) and brassinoteroids (BR), mostly as an indirect effect via auxin. Jasmonic acid also regulates root gravitropism through affecting the biosynthesis of auxin. It directly influences the gradient formation by modulating its polar distribution of auxin (Singh et al., 2017). JA induced gene expression is most characteristic in the outer layers of the roots (Gasperini et al., 2015).

The monolignol biosynthesis primarily regulated by Cinnamoyl CoA reductases plays an essential role in cell wall lignification in the casparian strips of the root. In the analyzed genomic region of the durum chromosome 6A, there are five cinnamoyl reductase genes encoded, and additional genes (e.g., ABC transporter, laccase) related to monolignol biosynthesis were also found in the region. Drought conditions were reported to enhance the monolignol biosynthesis in the root elongation zone of the seedlings by the inhibition of the cell wall extensibility and root growth (Ma, 2007). Similarly, lignin-related phenolics biosynthesis was also reported during biotic stress (Silva et al., 2010). It is plausible to therefore suggest that these candidate genes may be having a role to play in the constitiution of the durum root ideotype.

The concentration of genes functioning in fatty acid metabolism, monolignol biosynthesis and jasmonic acid biosynthesis pathways in the identified QTL region highlight the importance of jasmonic acid-auxin crosstalk in gravitropism perception and primary root angle formation. The potential target genes include enzymes involved in cell wall expansion (monolignol biosynthesis) and JA biosynthetic pathways. JA signaling in young root tissues acts contrary to auxin signaling effects, is also related to gravitropism and therefore might be directly related to root angle variations. Genetic variations observed in the encoding genes or their regulating *cis*-promoter elements can help to identify phenotypes where the coordinated negative impact of increasing JA levels and lignification is controlled. Next to the growth-related functions, these genes are also involved in abiotic and biotic stress responses, including drought stress.

### The Context-Dependency of Root System Ideotypes in Different Environments

The architecture of roots has great importance for sourcing underground water and nutrients which is essential for plant growth, particularly in marginal environments characterized by water limitation (Manschadi et al., 2006; Asif and Kamran, 2011). In barley, it has been hypothesized that shallow root growth as characterized by a wide root growth angle may be advantageous for accessing nutrients in the upper soil surface under environments where plants experience sporadic rainfall throughout the growing season (Robinson et al., 2016). However, studies showed that this may not be always the case. For example, in the Mediterranean climate of South Australia, which experiences high in-season precipitation, narrow root angle seems advantageous and tends to be associated with higher grain yield (McDonald, 2010). On the other hand, ‘steep, deep, and cheap’ ideotypes with longer roots or more root branching at depth are most desirable for enhanced access to nutrients and water stored in deep layers of the soil under environments experiencing terminal drought (Manschadi et al., 2008; Christopher et al., 2013; Lynch, 2013). Moreover, deep roots could be ideal to reduce between-plants competition for resources under high-density planting in high-input conditions (Manske and Vlek, 2002). Plants that express drought-adaptive traits under water-limited environments have been shown to sustain increased yield (Manschadi et al., 2010). This is likely due to increased water access post-anthesis which can be through deeper and more efficient root systems (Mace et al., 2012). Manschadi et al. (2006) demonstrated in their modeling study a yield increase of an extra 55 kg/ha for each millimeter of water extracted from the soil after anthesis and during the grain filling stage. The key reason for that was an increase in marginal water use efficiency to almost three times after anthesis, due to enhanced access to water available in deep soils (Kirkegaard et al., 2007; Christopher et al., 2013). The *qSRA-6A* QTL identified in this study is highly associated with root growth angle. This suggests deployment of the narrow (hap1) allele for *qSRA-6A* could be beneficial in breeding programs targeting production environments with deep soils that often experience water stress. The root plasticity under drought suggests that durum genotypes carrying the narrow allele may not have a yield penalty in high rainfall seasons because root growth appears to respond and take advantage of resource availability in the upper soil layers. The G × E for root development and utility of this feature should be further explored.

Interestingly, no association between *qSRA-6A* QTL with root and shoot biomass was found when comparing contrasting haplotype groups with similar genetic backgrounds. This implies that root growth angle and root biomass are under separate genetic control, opening up the possibility to create customized root systems, e.g., by using marker-assisted introgression approaches. Results of our study also highlighted that the ‘narrow-high’ ideotype produced the highest root proliferation at the deepest soil level with the smallest MXA under drought. This suggests the mechanism for accumulation of root biomass may not only be related to root branching at depth but also associated with an adaptive mechanism involving reduced water use uptake during early stages of crop development. If the loci controlling root biomass are deployed with loci influencing the direction of root growth, root proliferation could be directed and concentrated at desired soil depths. Such allelic combinations assembled through plant breeding could give rise to improved commercial varieties with designer roots tailored for specific target environments (Voss-Fels et al., 2018b). A similar observation was made in a rice study where a major RSA gene called *DEEPER ROOTING 1* (*DRO1*) was cloned (Uga et al., 2013). They showed that *DRO1* is involved in gravitropic response of root cells, thereby influencing root growth direction, but without a significant effect on root biomass. The *qSRA-6A* QTL is unlikely to be *DRO1* because the ortholog is located on the group 5 chromosomes of wheat and wild emmer. Additional QTL related to root growth angle (*DRO2*, *DRO3*, *DRO4*, and *DRO5*) have also been reported (Kitomi et al., 2015). The QTL region *DRO4* is located on the long arm of chromosome 2 in rice and *Aux/IAA8*, *OsPIN1*, and *SAUR* were reported as major contributors to the measured phenotypes (Kitomi et al., 2015; Lou et al., 2015). Using these rice orthologs we found that the corresponding genes are not overlapping with the genomic region determined in our study and are located closer to the centromere of chromosome 6A both in hexaploid and tetraploid wheat. Recently, another QTL has been reported by Wang et al. (2018), showing a significant role of *OsPIN2* gene in root growth angle in rice. *OsPIN2* encodes an Auxin-efflux carrier (Os06g0660200) that showed the highest sequence homology to the homologous gene group on chromosome 7 in wheat (TraesCS7A02G492400, TraesCS7B01G398100, and TraesCS7D01G478800). Overall, the lack of alignment between known genes in rice and the QTL on 6A mapped in the present study implies the region likely contains novel or currently uncharacterized gene(s).

The genetically stable effects of the *qSRA-6A* haplotypes across three different families implies that marker-assisted backcrossing strategies using the marker sequences identified in this study could effectively modulate RSA in future breeding attempts. Numerous studies have reported that root growth angle at the seedling stage was predictive for root growth angle in the field (Tuberosa et al., 2002a; Landi et al., 2010; Li et al., 2015; Richard et al., 2015; Uga et al., 2015; Kanehisa et al., 2016). Furthermore, recent studies have shown that RSA can be manipulated through recurrent phenotypic selection at the seedling stage under glasshouse conditions in which root traits could be measured at high broad-sense heritabilities (ranging from 0.62 to 0.79), leading to significant shifts in population distributions after a few cycles of selection (Alahmad et al., 2018; Richard et al., 2018). This offers plant breeders different options to directly manipulate RSA.

One major limitation for the direct consideration of root traits in defined breeding goals is the high context-dependency of varying RSA in different environments and the interplay of roots with other key phenology traits like flowering time in the expression of the end-point trait such as grain yield (Voss-Fels et al., 2018b). It was recently shown in a comprehensive study involving multi-environment trials in barley that the genetic correlation of root growth angle and yield was highly context dependent, ranging from situations in which shallow roots were associated with increased yield performance and vice versa (Robinson et al., 2018). Multi-environment field trials are required to thoroughly evaluate the value of *qSRA-6A* and different root ideotypes to improve or stabilize durum grain yield in a range of environmental circumstances.

## Author Contributions

SA and LH conceived and designed the experiments. SA, CY, AA, and GQ performed the experiments. SA, ED, CY, KV-F, EM, and AJ analyzed the data. SA, JA, FB, and LH provided germplasm. SA, KEH, ED, CY, EM, AJ, KV-F, JA, FB, JC, and LH wrote or reviewed the manuscript. All authors read and approved the final manuscript.

## Conflict of Interest Statement

The authors declare that the research was conducted in the absence of any commercial or financial relationships that could be construed as a potential conflict of interest.

## Footnotes

1 http://imagej.nih.gov/ij/

2 http://popart.otago.ac.nz

3 https://github.com/plantinformatics/pretzel

4 https://software.broadinstitute.org/morpheus
