# Supplementary material for: A Major Root Architecture QTL Responding to Water Limitation in Durum Wheat
Source: Front Plant Sci. 2019 Apr 10;10:436. doi: 10.3389/fpls.2019.00436 (PMC6468307; doi:10.3389/fpls.2019.00436)

**Supplementary Figure 1.** Comparison of four root ideotypes grown in root growth rhizoboxes under drought and well-watered conditions five weeks after sowing. (A) Differences in the percentage of root distribution between drought and well-watered conditions at different depths of the root rhizobox; upper-layer of the soil 0-20 cm, mid-layer of the soil 20-40 cm, deep-layer of the soil 40-60 cm. Visualization of root distribution for the four ideotypes (B) wide-low ideotype, (C) wide-high ideotype, (D) narrow-high ideotype, (E) narrow-low ideotype at different depths under drought condition.


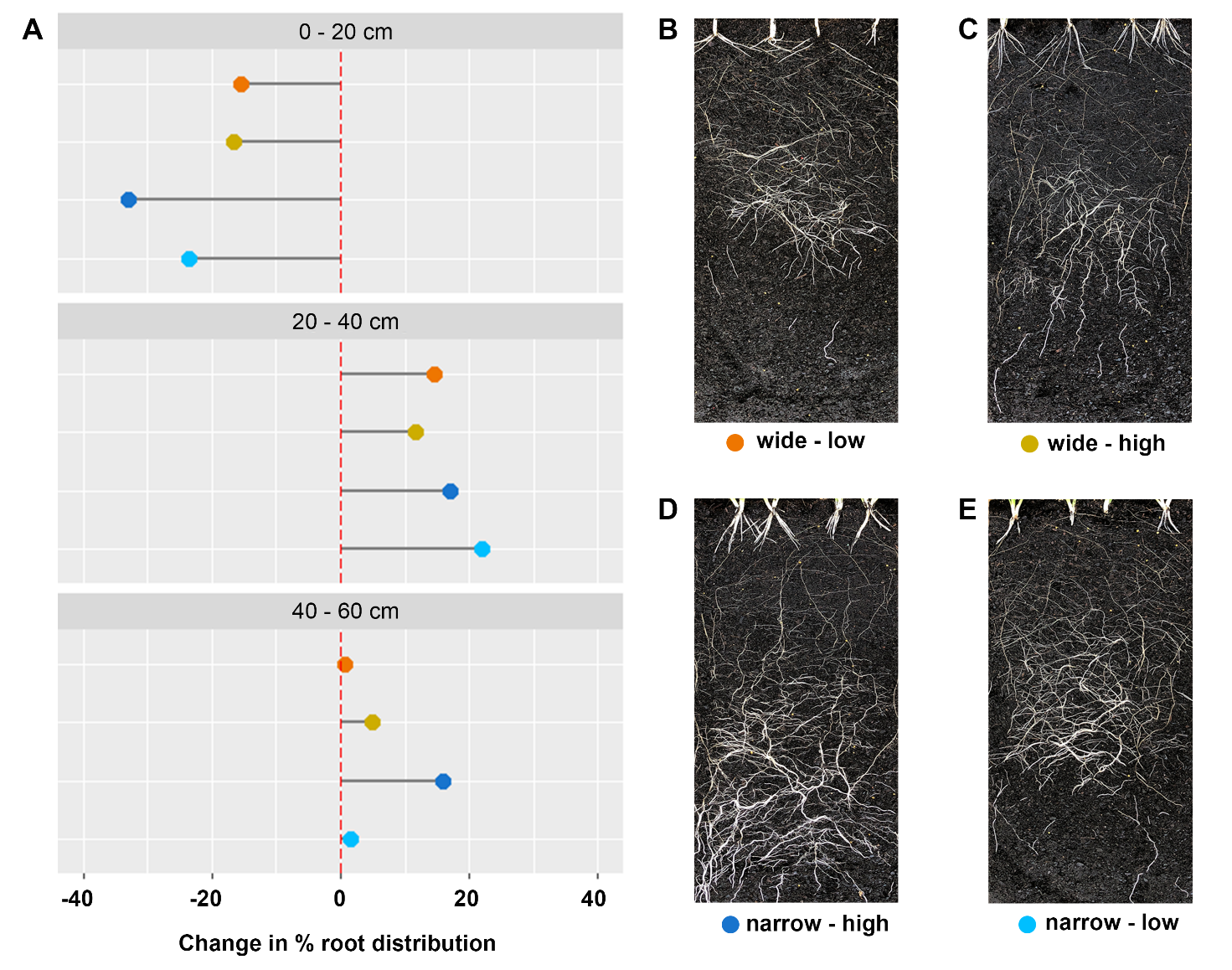


**Supplementary Figure 2.** Gravimetric soil moisture levels (%) over time at 50 cm depth of the rhizobox (bottom section 40-60 cm). The color of the line corresponds to the treatments: well-watered (blue) drought (red). Error bars are the standard error of the mean of 12 replicates per treatment. Means with the same letters are statistically similar.


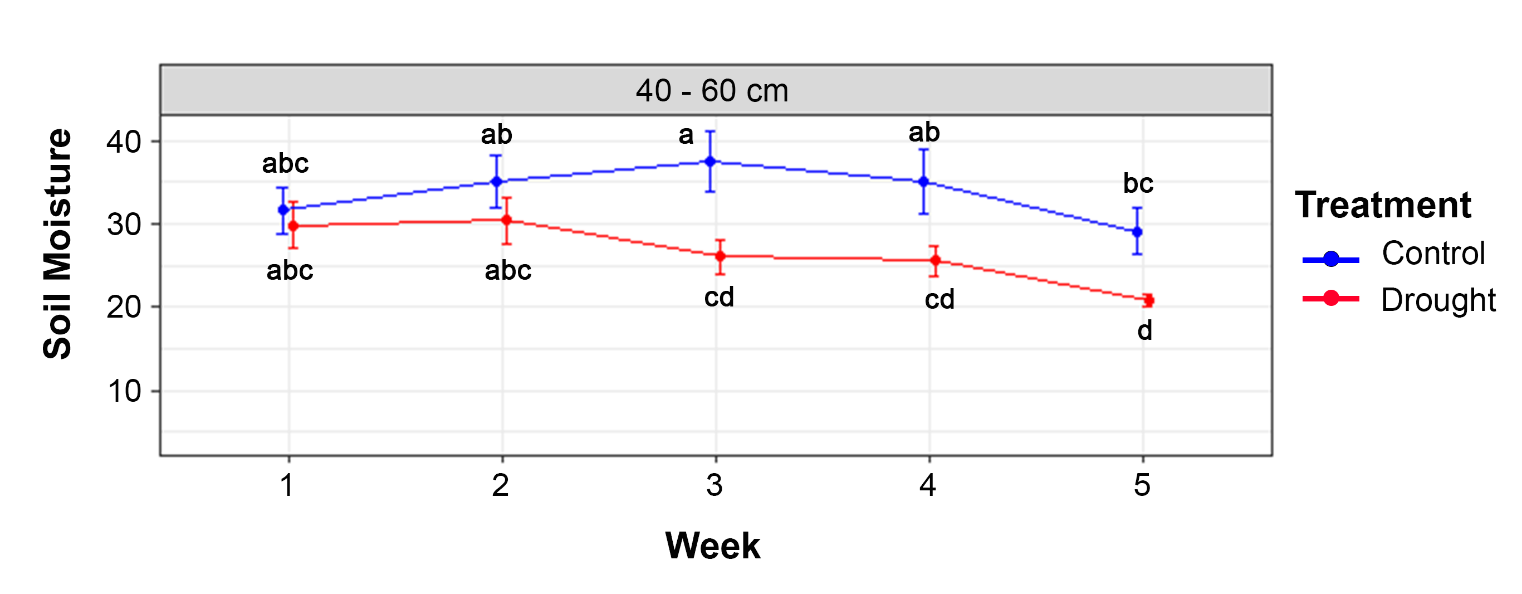


**Supplementary Figure 3.** Collinearity analysis of the root angle QTL region at the distal end of Chromosome 6A in the *T. durum* and *T. aestivum* reference genomes. Blue lines represent collinearity of the identified QTL peak markers. Deep red represents genes that show high expression levels in early development stages in roots. Green coloring represents genes with root-specific expression in the later stage.


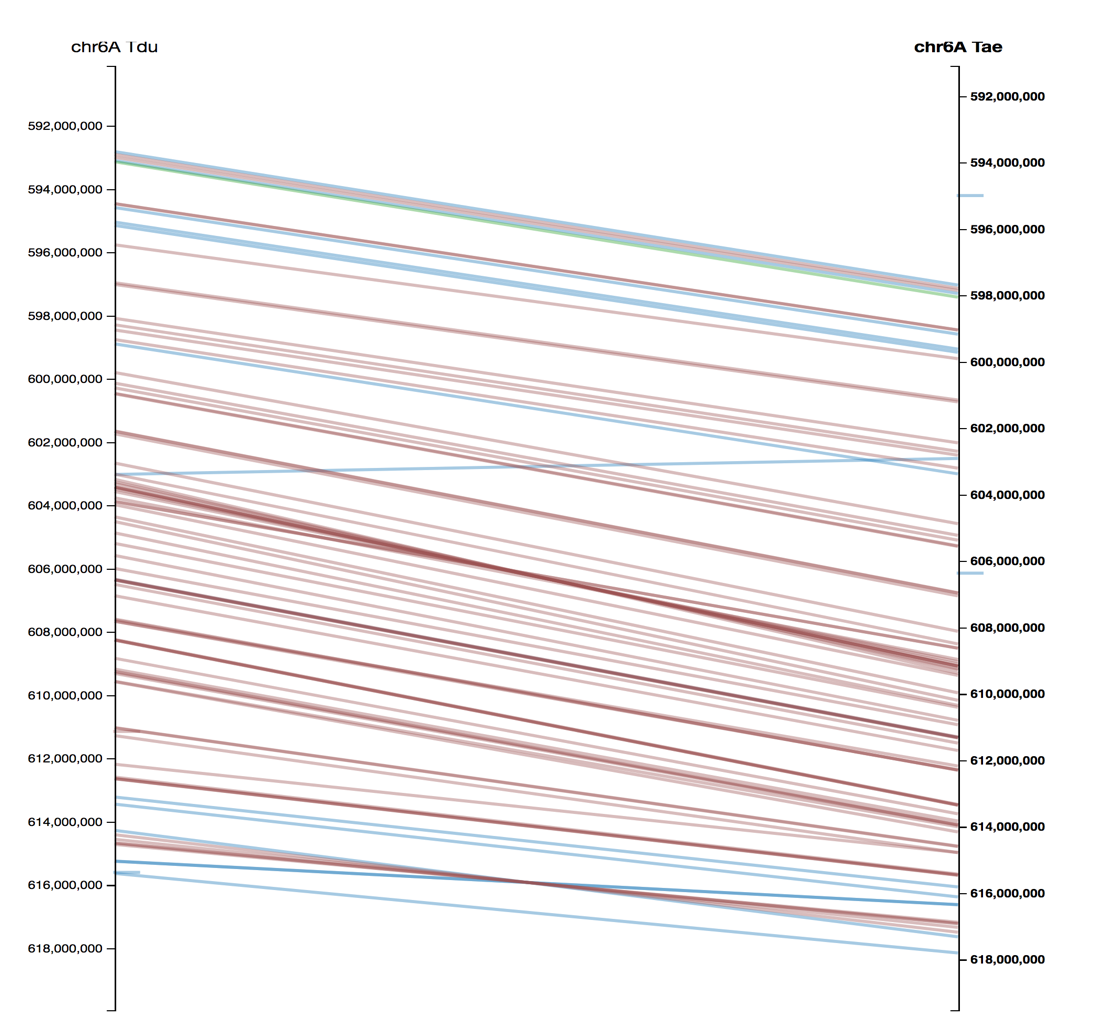

Supplement: Supplementary file 1 [file Data_Sheet_1.docx]
